# Complete Genome Sequence of *Desulfovibrio piger* FI11049

**DOI:** 10.1128/genomeA.01528-16

**Published:** 2017-02-16

**Authors:** Udo Wegmann, Carmen Nueno Palop, Melinda J. Mayer, Emmanuelle Crost, Arjan Narbad

**Affiliations:** Gut Health and Food Safety Programme, Institute of Food Research, Norwich, United Kingdom

## Abstract

The complete genome sequence of *Desulfovibrio piger* FI11049 was determined. The genome consists of a single circular chromosome of 2,807,531 bp encoding seven rRNA operons, 76 tRNA genes, and 2,535 coding genes.

## GENOME ANNOUNCEMENT

The human gastrointestinal tract hosts a plethora of resident microorganisms with bacterial cell densities in the colon reaching 10^11^ cells per gram of content (1). Intestinal microbiomes contribute considerably to the health of their human hosts by, for example, providing essential vitamins and by breaking down and fermenting dietary fiber into short-chain fatty acids (SCFAs), a role that falls to the primary fermenters residing in the large intestine (e.g., members of the *Firmicutes* and *Bacteroidetes* phyla), which, besides supplying energy, has a wider physiological impact on the host. During the fermentation of dietary fibers to SCFAs, many primary fermenters also produce molecular hydrogen (H_2_) by using H+ as an electron acceptor in order to regenerate NAD^+^. If H_2_ levels within the large intestine were left to increase unchecked, the ability of primary fermenters to regenerate NAD^+^, and in turn grow, would be impaired (2). Therefore, H_2_-consuming microbes such as acetogens, methanogens, and sulfate-reducing bacteria (SRB), like *Desulfovibrio piger*, play an essential role in maintaining the metabolism of primary fermenters. However, SRB have been suspected to contribute to gastrointestinal disease (3) due to the production of hydrogen sulfide, which can be considered toxic to the gut epithelium (4) and has been shown to be genotoxic to mammalian cells (5). *D. piger*, an SRB from the *Proteobacterium* phylum (deltaproteobacterium) and formerly known as *Desulfomonas pigra*, is a Gram-negative, nonmotile, rod-shaped bacterium 0.8 to 1.3 μm ×1.2 to 5 μm in size. In a study involving fecal samples of 34 healthy individuals, *D. piger* was shown to be the most frequent SRB, present in 60% of samples (6). In another study highlighting its potential involvement in inflammatory bowel diseases (IBDs), its prevalence was shown to be significantly higher in IBD patients compared to healthy individuals or non-IBD patients (7). *D. piger* FI11049 was isolated from the feces of an ulcerative colitis patient, and its complete genome sequence can aid in resolving the question of whether SRB, and *D. piger* in particular, are a contributing factor in gastrointestinal diseases. To our knowledge, this is the first complete genome sequence of this species.

The complete genome sequence was determined using the Illumina GAIIx platform. The paired-end data were assembled with Abyss (8), resulting in 88 contigs. Multiplex PCR was employed to identify adjoining contigs and respective primer pairs for which no linkage had been established previously. Standard PCR, followed by primer walk sequencing on the resulting products, was used to close the gaps. In addition, data from one PacBio RSII SMRT cell assembled with HGAP.3 were obtained. The final sequence assembly of the different sequencing platform outputs was carried out with the Staden package (9). The finished *D. piger* FI11049 sequence was annotated using the RAST server (10).

The genome consists of a single circular chromosome of 2,807,531 bp with an average GC content of 64.18%. It encodes seven rRNA operons, 76 tRNA genes, and 2,535 coding genes.

### Accession number(s).

The genome sequence has been deposited at the European Nucleotide Archive under the accession number LT630450.
